# Chemical profiling and cytotoxicity screening of agarwood essential oil (*Aquilaria sinensis*) in brine shrimp nauplii and cancer cell lines

**DOI:** 10.1371/journal.pone.0310770

**Published:** 2024-11-07

**Authors:** Sook Wah Chan, Valizadeh Lakeh Mahmoud, Xin Wang, Ming-Li Teoh, Kar Min Loh, Chun Howe Ng, Won Fen Wong, Chung Yeng Looi

**Affiliations:** 1 Faculty of Health and Medical Sciences, School of Biosciences, Taylor’s University, Subang Jaya, Selangor, Malaysia; 2 Food Security & Nutrition Impact Lab, Taylor’s University, Subang Jaya, Selangor, Malaysia; 3 Clean Technology Impact Lab, Taylor’s University, Subang Jaya, Selangor, Malaysia; 4 Faculty of Medicine, Department of Medical Microbiology, Universiti Malaya, Kuala Lumpur, Malaysia; 5 Digital Health and Medical Advancements Impact Lab, Taylor’s University, Subang Jaya, Selangor, Malaysia; University of Milan, ITALY

## Abstract

Agarwood essential oil (AEO) has gained attention from healthcare industries due to its numerous pharmacological properties. However, a comprehensive understanding of the chemical composition and its cytotoxic property is lacking. The objective of this study was to investigate the chemical profile as well as the cytotoxic concentration range of AEO derived from *Aquilaria sinensis* agarwood. Gas chromatography-mass spectrometry (GC-MS) was employed to identify the AEO components. Results showed that sesquiterpenes and sesquiterpenoids constitute 95.85% of the AEO. Among the major compounds identified are *allo*-aromadendrene (13.04%), dihydro-eudesmol (8.81%), α-eudesmol (8.48%), bulnesol (7.63%), τ-cadinol (4.95%), dehydrofukinone (3.83%), valerenol (3.54%), cis-nerolidol (2.75%), agarospirol (2.72%), dehydrojinkoh-eremol (2.53%), selina-3,11-dien-9-al (2.36%), guaiol (2.12%) and caryophyllene oxide (2.0%). The presence of volatile quality marker compounds such as 10-epi-ϒ-eudesmol, aromadendrane, β-agarofuran, α-agarofuran, γ-eudesmol, agarospirol and guaiol, with no contaminants detected, indicates that the extracted AEO is of high purity. Interestingly, the AEO displayed moderate to high toxicity in brine shrimp lethality test (BLST). All studied tumor cell lines (MDA-MB-231, HepG2, B16F10) exhibited varying degrees of sensitivity to AEO, which resulted in time and dose-dependent reduction of cell proliferation. Moreover, flow cytometry analysis revealed that AEO could induce apoptosis in treated HepG2 cells. Our findings showed that AEO contains bioactive components that may be exploited in future studies for the development of anti-cancer therapeutics.

## Introduction

Cancer is a pathological condition caused by uncontrolled cell proliferation, which is a hallmark of oncogenesis and progression [1]. According to the latest issue of the International Agency for Research on Cancer (IARC) in 2020, breast cancer in women has now become the most frequently diagnosed cancer, surpassing lung cancer. Approximately 2.3 million new cases, accounting for 11.7% of diagnoses, were reported, followed by lung (11.4%), colorectal (10.0%), prostate (7.3%) and stomach cancer (5.6%). Despite this shift, lung cancer remains the leading cause of cancer-related deaths, with an estimated 1.8 million fatalities (18%). Colorectal cancer stands at 9.4%, followed by liver (8.3%), stomach (7.7%) and breast cancer (6.9%) [2]. Among men, lung cancer is the leading cause of mortality [3]. The primary cause of cancer-related death in women is breast cancer [3]. On the other hand, acute lymphoblastic leukemia is the top hematological cancer in children and adolescents, representing 20% of all cancers diagnosed in persons aged < 20 years [4].

Depending on the type of cancer and how advanced it is, the standard treatments are surgery, chemotherapy and radiation. However, resistance to radiotherapy and chemotherapy may result in treatment failure or even cancer recurrence [5]. Thus, the development of new therapeutic agents to overcome these pressing issues is necessary. Chemotherapy is an important component of modern cancer therapy, which has evolved over centuries. While the historical use of plants in medicine dates back to the dawn of civilization, the World Health Organization (WHO) reports that people in Asia and some parts of Africa widely rely on herbal remedies for disease management [6]. At present, a significant amount of modern pharmaceutical drugs, particularly those used in cancer therapy, are derived from natural ingredients. Phytochemicals like vincristine, camptothecin, and paclitaxel have emerged as important as well as reliable chemotherapeutic agents in cancer treatment [7]. This long-standing reliance on natural sources highlights the ongoing pursuit of efficacious therapeutic agents sourced from plants.

**Fig 1:**
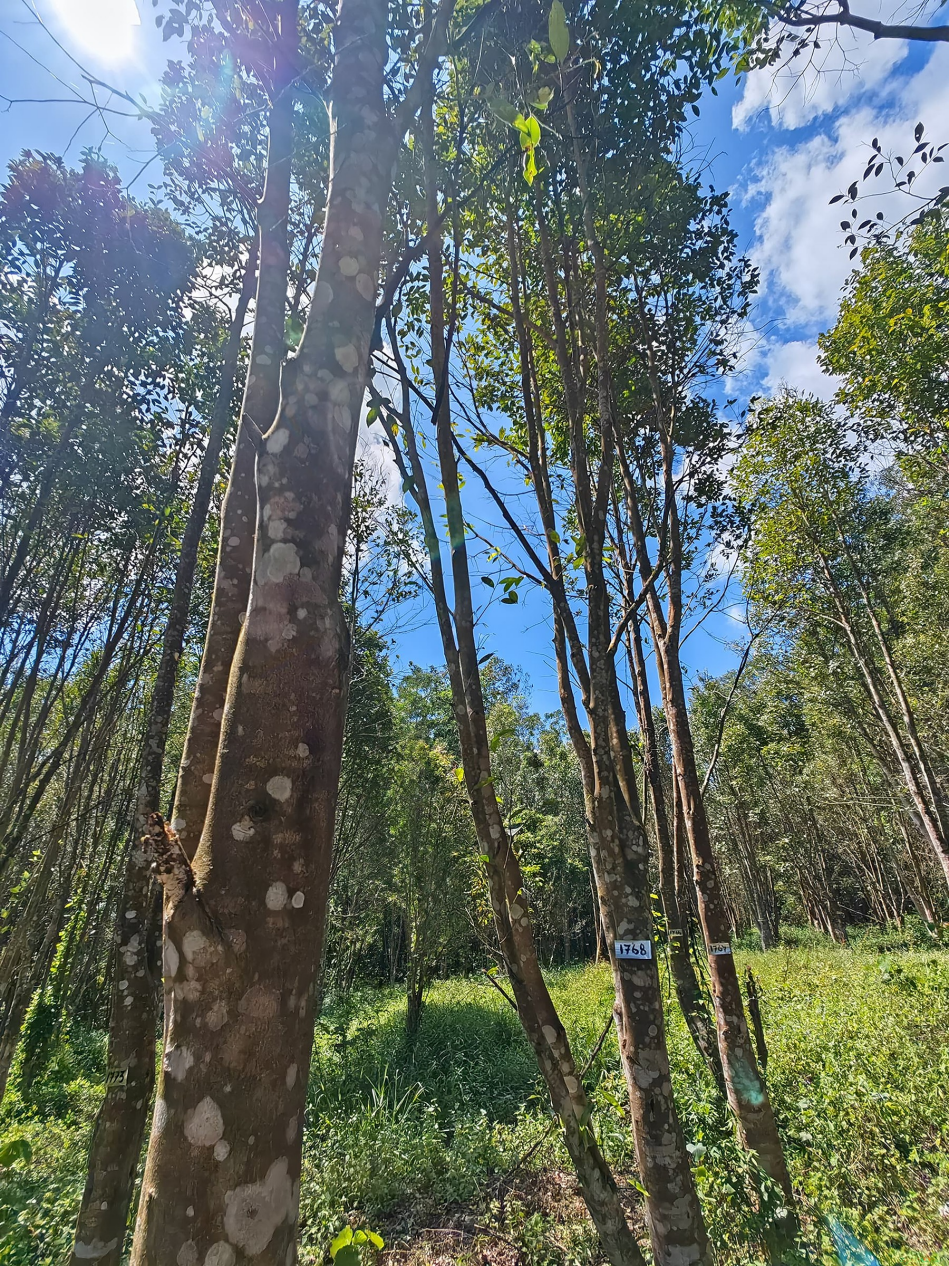
Cultivated agarwood (*A*. *sinensis*) tree.

Agarwood, also known as eaglewood, oud, aloeswood, and gaharu is a highly valuable fragrant heartwood used for a wide range of non-timber purposes in perfume, cosmetics industry, medicinal preparations as well as for religious ritual purposes [8]. The genus *Aquilaria* or simply known as agarwood from the family Thymelaeaceae, has different species including *crassna*, *malaccensis*, and *sinensis* (Fig 1). Agarwood is well known for its resinous secretion against injuries and this tree is commonly found in Southeast Asian countries and Southern China. Agarwood is rich in bioactive compounds, including sesquiterpenes, chromones, flavonoids, benzophenones, diterpenoids, triterpenoids and lignans, which show myriad pharmacological effects [9–11]. A number of studies reported that these chemicals extracted from *Aquilaria* wood exhibit acetylcholinesterase inhibition, anti-fungal, anti-bacterial, anti-inflammatory, anti-cancer and analgesic actions [12–14].

Previous study indicates that plant-derived essential oil possesses anti-tumor potential against leukemia as well as cancers of the mouth, breast, lung, prostate, liver, and colon [15]. Study revealed that specific components of essential oils might increase the cytotoxic action of chemotherapy drugs, such as docetaxel, paclitaxel, and 5-fluorouracil, on different cancer cell lines. This suggests that the dosage of these drugs may be reduced while still having the same effect [16]. Till date, the cytotoxic and chemical profile of AEO derived from *A*. *sinensis* is understudied, which hampered its feasibility to be used for potential medication or therapeutic agents. Hence, the present study was conducted to characterize the chemical constituents of AEO via GC-MS and to evaluate its cytotoxic profile using brine shrimp (*Artemia salina)* and cancer cell lines (MDA-MB-231, B16F10, HepG2).

## Materials and methods

### Plants material

**Fig 2:**
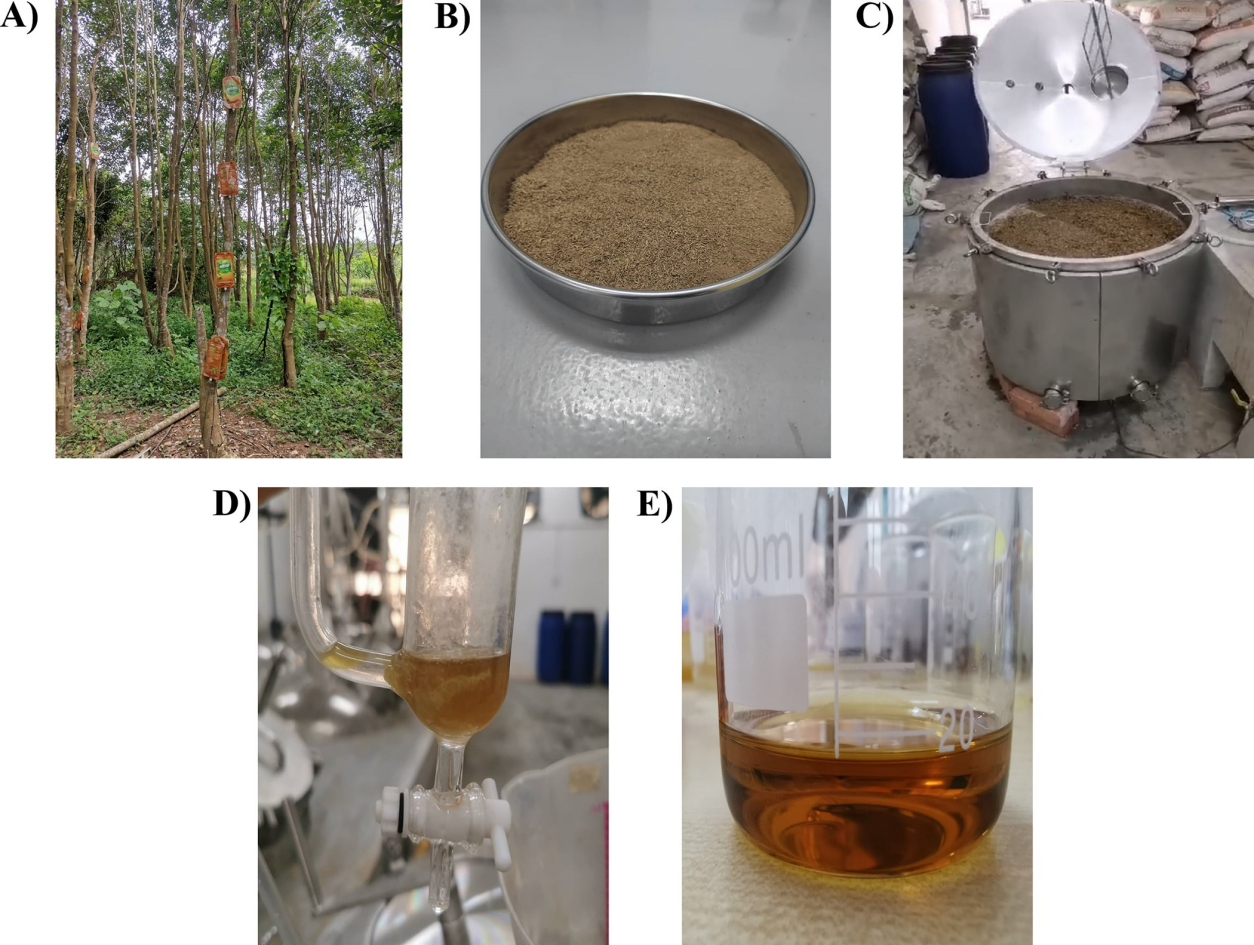
Illustrations of (**A**) Cultivated agarwood (*A*. *sinensis*) plantation; (**B**) Agarwood powder; (**C**) Agarwood powder in distillation tank; (**D**) AEO collected at the bottom of the separation funnel; (**E**) Final AEO.

Agarwood, the resinous heartwood from *A*. *sinensis* was harvested from a cultivated agarwood plantation in Tangkak, Johor, located at a longitude of 2.2951° N and latitude of 102.5756° E, which is registered with the Malaysian Timber Industry Board (MTIB) (Fig 2A). Taxonomic identification was performed by Forest Research Institute of Malaysia (FRIM). Voucher specimen (202312–1) is deposited at the Lab 2, Department of Medical Microbiology, Faculty of Medicine, Universiti Malaya.

### Extraction of agarwood essential oil

The agarwood was separated from the white wood, crushed into smaller pieces, and air-dried under the sun for 1–2 days until completely dry. Subsequently, the dried agarwood dust was ground into powder (Fig 2B) and stored in a bag at room temperature for further extraction. Before the extraction process, 20 kg of agarwood powder underwent a 14-day soaking period in filtered water to facilitate the release of AEO. The soaked powder and 200 L of filtered water were then introduced into a 250 L stainless steel tank of a novel designed industrial distiller (Lian Sin Enterprise Sdn. Bhd., Selangor, Malaysia) (Fig 2C) at a sample:water ratio of 1:10 for optimal yield and quality. The mixture was heated to a boil at 100°C, generating steam containing both water and AEO. The steam was condensed and collected in an essential oil separation funnel. The excess condensed water was recycled back to the distillation tank during the extraction process. After 120 h of hydro-distillation, the water will be removed, followed by the retrieval of the floating AEO through the bottom of the separation funnel, as illustrated in Fig 2D. The AEO was obtained as a pale yellow liquid (Fig 2E).

### Chemical profiling by GC-MS

The volatile constituents of the sample were analyzed using a gas chromatography system, the Agilent 7890B (Agilent Technologies Inc., Santa Clara, CA, USA) coupled with an Agilent 5977A quadrupole mass spectrometer and Agilent DB-1ms capillary column (30 m × 0.25 mm x 0.25 μm film thickness) based on the method adopted from Eissa et al. [17] with modifications. GC was performed using the following conditions: autosampler injection (1 μL); injection temperature (230°C), carrier gas (helium) flow rate (1 mL/min); programmed oven temperature, 70°C held for 3 min, ramped from 70 to 230°C at a rate of 3°C/min, held for 3 min. The detector temperature was 240°C. The detected peaks from the total ion chromatography (TIC) and mass chromatograms were identified based on the National Institute of Standards and Technology (NIST) 2020 mass spectral library. The RI value of each component was determined relative to the retention times (RT) of a series of C7-C20 n-alkanes with linear interpolation on the Agilent DB-1ms column. The peak area (%) of each individual component of the essential oil was expressed as the percentage of the peak area relative to the total peak area.

### Brine shrimp lethality test (BSLT)

The brine shrimp lethality test was performed according to the protocol of Asaduzzaman et al. [18]. The method estimates *in vivo* lethality in a simple zoological organism, using nauplii of the brine shrimp *Artemia salina*. Artificial seawater (ASW) was prepared by mixing 7.5 g of a commercially available salt mixture with 500 mL of distilled water according to the instructions. *A*. *salin*a eggs were added to a small commercial nauplius hatching tank and incubated in an ASW under a halogen lamp providing direct light and heat. The hatched shrimp was allowed to mature for 24 h as nauplii. Hatched shrimps were attracted to light, and brine shrimp eggless nauplii were collected from illuminated sections of the aquarium. Ten nauplii were counted visually in the stem of a graduated Pasteur pipette against an illuminated background and transferred to a test tube. A stock solution (50 mg/mL) is prepared by dissolving the AEO in saline containing 15% propylene glycol and 1% Tween 80. This can be used to serially dilute it to produce a range of concentrations 31.25, 62.5, 125, 250, 500, 1000 μg/mL. Solvent alone was used as negative control. Testing was performed with three replicates for each dosage and 10 nauplii per replicate. Each test tube was illuminated for 24, 48 and 72 h. Survivors were counted visually by two independent enumerators. The LC_50_ values were determined with probit analysis using the Statistical Package for the Social Sciences (SPSS) application.

### Cell culture

Human breast adenocarcinoma (MDA-MB-231), hepatocellular (HepG2) and murine melanoma (B16F10) cell lines were purchased from American Type Culture Collection (ATCC). The cells were grown in Dulbecco’s Modified Eagle’s Medium (DMEM) supplemented with 10% FBS and 1% penicillin-streptomycin and incubated at 37°C, 5% CO_2_ as described previously [19].

### Cell viability assay

MTT assay was performed to measure cell viability, according to previous protocol [20]. The cells were seeded in 100 μL of complete culture media, at a density of 5 × 10^4^ per mL in 96 well plates. The seeded cells were incubated for 12 h at 37°C and 5% CO_2_. The cells were then treated with AEO at different concentrations including 0.975, 1.95, 3.91, 7.81, 15.625, 31.25, 62.5, 125, 250, 500 μg/mL. A total of 20 μL of MTT solution was added to each well after the cells were treated with AEO for the indicated time point. After 3 h of incubation, the media was discarded and 100 μL of DMSO was added to each well. The absorption of produced formazan was measured at 570 nm using a BioTek EPOCH 2 microplate reader. The cell viability was calculated as following:

Cellviability(%)=(ODSample/ODControl)×100

### Apoptosis assay

To quantify the percentages of apoptosis versus necrosis cell death, cells were seeded at 1×10^5^ per ml on 25 cm^2^ flask overnight before treated with control solvent (15% propylene glycol and 1% Tween 80) and AEO at various concentrations (50 μg/mL and 100 μg/mL) for 16 h. Determination of apoptotic/necrotic cells by fluorescent staining was done as described previously [21]. Briefly, cells were incubated with FITC-annexin V and propidium iodide (PI) (BD Biosciences) in a binding buffer for 15 minutes in the dark. Stained cells were immediately subjected to flow cytometry analyses using Novocyte flow cytometer system (Agilent).

### Statistical analysis

The statistical analysis was performed using GraphPad Prism 9.0. Two-way analysis of variance (ANOVA) was performed to determine the significant differences between means at a 95% confidence interval (*p* < 0.05).

## Results and discussion

### Yield, smells, and appearance of AEO

The agarwood used in this study originated from *A*. *sinensis* cultivated in Tangkak, Johor (Fig 2A). By adopting the ITS2, matK, and rbcL barcode sequences, the agarwood sample was identified as *A*. *sinensis*. The AEO was extracted from agarwood (*A*. *sinensis*) powder using hydro-distillation (HD) (Fig 2C). HD is the most conventional method employed by industries for the extraction of essential oil from agarwood using water, making it a relatively safe and environmentally-friendly process [22,23]. The AEO obtained had a clear, brown hue with a slightly golden tint and emitted a strong characteristic woody scent, accompanied by hints of bitter, sweet, and spicy aromas (Fig 2E).

The average yield of AEO through HD for 120 h at 100°C was reported as 1 g/kg of dried agarwood powder, equivalent to 0.1% (v/w). The yield of AEO in this experiment was similar to that of resinous heartwood and agarwood leaves reported in the previous literature. Sulaiman et al. [24] reported that the maximum extraction yield of AEO from *A*. *malaccensis* was 0.18% using the HD method for 72 h at 100°C. Other research done by Wu et al. [25] investigated the extraction of leaf aromatic oils from *A*. *sinensis* through HD, with a reported yield of 0.1% at 100°C for 5 h. Despite its practicability and safety, HD remains a time-consuming and low efficiency extraction method. In this work, soaking agarwood powder as a pre-treatment was performed to enhance the swelling of the cell wall of agarwood, thereby releasing more AEO [26].

Several new extraction methods, including solvent extraction (SE), enzyme-assisted extraction (EAE), microwave-assisted extraction (MAE), ultrasound-assisted extraction (UAE), and supercritical fluid extraction (SFE) have been explored for extracting AEO [27–29]. In Radzi et al. [30] study, agarwood that underwent microwave pre-treatment demonstrated a threefold increase in yield compared to conventional HD. On the other hand, SFE exhibited a significant increase in AEO yield, rising from 0.061% (obtained by HD) to 0.317% [31]. Nevertheless, these extraction methods are still not widely utilized in the agarwood industry due to the safety concerns (SE), high costs (EAE, SFE), and upscaling issues (MAE, UAE) [23].

### Chemical composition analysis

**Fig 3:**
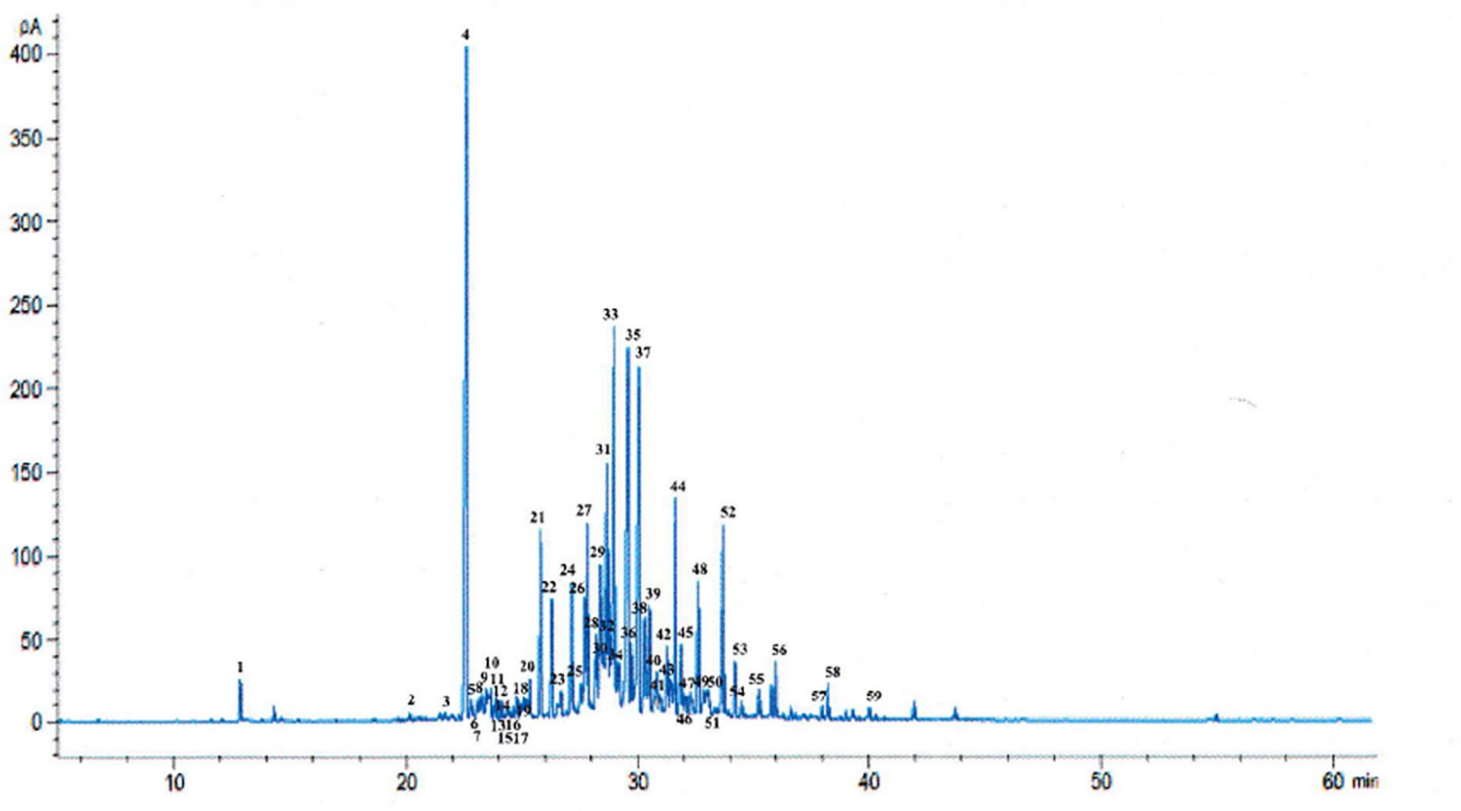
Chromatogram of the analysis by GC for AEO obtained from *A*. *sinensis*.

Profiling of chemical constituents in plant species is an important step for the investigation of their medical effects. The chemical constituents identified from the medicinal plants are the potential sources for drug development. As AEO is valued for its distinctive and pleasant fragrance, GC-MS is extensively employed to identify its compounds and assess its quality [32]. In this study, the AEO was eluted and analyzed by GC-MS using DB-1ms capillary column. The identification of the individual components of the AEO was based on the matching of their mass spectra with the NIST 2020 library. Fig 3 displays the GC-MS spectra of AEO, revealing the presence of 59 chemical compounds. The compounds were numbered in accordance with corresponding peaks. Visually, small peaks could be observed in large quantities, corresponding to the compounds that could not be determined (Fig 3). Chemical compounds showed up before retention time (RT) of 25.3 min were mainly sesquiterpenes and sesquiterpenoids. Most of the sesquiterpenoids and other compounds were detected between 25.3 and 40.0 min.

The chemical compounds found in AEO are listed in Table 1 according to their sequence of elution on a DB-1ms capillary column. A summary is provided in Table 1 for the distribution of chemical classes, retention time, % contents of the constituent components, as well as CAS number. Overall, the total percentage of sesquiterpenoids was the highest at 77.36%, followed by sesquiterpenes (18.49%) and other compounds (1.41%). This finding is in good agreement with previous studies which found sesquiterpenoids as the major component in AEO [33–35]. However, the amount of sesquiterpenoids varied depending on the agarwood species, origin, and extraction methods. Other studies found varying amounts of sesquiterpenoids, ranging from 63.87% (India, *A*. *malaccensis*) to 67.40% (Malaysia, *A*. *malaccensis*) and 49.91% (Thailand, *A*. *crassna*) [36]. The findings in Tian et al. [37] showed that sesquiterpenoids were present in AEO (*A*. *sinensis*) at 68.68% through steam distillation, whereas the supercritical extraction method yielded sesquiterpenoids at 23.78%.

**Table 1:** Chemical compounds identified in AEO obtained from *A*. *sinensis*.

No	Compounds	Molecular formula	Retention indices (RI)	Retention time (min)	Peak area (%)	Phytochemical groups	CAS no
1	4-Phenyl-2-butanone	C_10_H_12_O	1207	12.841	0.53	Other compounds	2550-26-7
2	α-Gurjunene	C_15_H_24_	1397	20.132	0.10	Sesquiterpene	489-40-7
3	Aromadendrene	C_15_H_24_	1439	21.667	0.10	Sesquiterpene	489-39-4
4	*allo*-Aromadendrene	C_15_H_24_	1463	22.585	13.04	Sesquiterpene	25246-27-9
5	α-Curcumene	C_15_H_22_	1468	22.79	0.44	Sesquiterpene	644-30-4
6	γ-Selinene	C_15_H_24_	1474	23.032	0.28	Sesquiterpene	515-17-3
7	β-Agarofuran	C_15_H_24_O	1477	23.146	0.35	Sesquiterpenoid	NA
8	β-Vetispirene	C_15_H_22_	1480	23.259	0.52	Sesquiterpene	28908-27-2
9	Valencene	C_15_H_24_	1484	23.436	0.56	Sesquiterpene	4630-07-3
10	α-Selinene	C_15_H_24_	1488	23.574	0.55	Sesquiterpene	473-13-2
11	Dihydro-β-agarofuran	C_15_H_26_O	1489	23.642	0.46	Sesquiterpenoid	5956-09-2
12	δ-Guaiene	C_15_H_24_	1495	23.874	0.54	Sesquiterpene	3691-11-0
13	γ-Cadinene	C_15_H_24_	1500	24.08	0.26	Sesquiterpene	39029-41-9
14	α-Elemene	C_15_H_24_	1508	24.35	0.28	Sesquiterpene	5951-67-7
15	δ-Cadinene	C_15_H_24_	1511	24.461	0.12	Sesquiterpene	483-76-1
16	Kessane	C_15_H_26_O	1515	24.598	0.17	Sesquiterpenoid	3321-66-2
17	Selina-3.7(11)-diene	C_15_H_24_	1524	24.925	0.34	Sesquiterpene	6813-21-4
18	α-Calacorene	C_15_H_20_	1527	25.051	0.30	Sesquiterpene	21391-99-1
19	Dehydro-aromadendrene	C_15_H_22_	1530	25.151	0.29	Sesquiterpene	NA
20	α-Agarofuran	C_15_H_24_O	1535	25.339	0.60	Sesquiterpenoid	5956-12-7
21	*cis*-Nerolidol	C_15_H_26_O	1547	25.779	2.75	Sesquiterpenoid	142-50-7
22	*nor*-Ketoagarofuran	C_14_H_22_O_2_	1560	26.264	1.75	Sesquiterpenoid	5986-25-4
23	β-Vetivenene	C_15_H_22_	1571	26.668	0.77	Sesquiterpene	27840-40-0
24	Caryophyllene oxide	C_15_H_24_O	1582	27.115	2.00	Sesquiterpenoid	1139-30-6
25	Tetradecanal	C_14_H_28_O	1593	27.521	0.54	Other compounds	124-25-4
26	Guaiol	C_15_H_26_O	1597	27.697	2.12	Sesquiterpenoid	489-86-1
27	10-*epi*-γ-Eudesmol	C_15_H_26_O	1601	27.823	3.07	Sesquiterpenoid	15051-81-7
28	γ-Eudesmol	C_15_H_26_O	1612	28.208	1.42	Sesquiterpenoid	1209-71-8
29	Agarospirol	C_15_H_26_O	1617	28.379	2.72	Sesquiterpenoid	1460-73-7
30	Hinesol	C_15_H_26_O	1620	28.475	1.41	Sesquiterpenoid	23811-08-7
31	τ-Cadinol	C_15_H_26_O	1625	28.676	4.95	Sesquiterpenoid	5937-11-1
32	β-Eudesmol	C_15_H_26_O	1629	28.801	1.14	Sesquiterpenoid	473-15-4
33	α-Eudesmol	C_15_H_26_O	1634	28.971	8.48	Sesquiterpenoid	473-16-5
34	Jinkoh-eremol	C_15_H_26_O	1640	29.174	1.21	Sesquiterpenoid	94201-17-9
35	Dihydro-eudesmol	C_15_H_28_O	1651	29.58	8.81	Sesquiterpenoid	6770-16-7
36	Kusunol	C_15_H_26_O	1655	29.716	1.07	Sesquiterpenoid	20489-45-6
37	Bulnesol	C_15_H_26_O	1664	30.044	7.63	Sesquiterpenoid	22451-73-6
38	Dehydrojinkoh-eremol	C_15_H_24_O	1671	30.297	2.53	Sesquiterpenoid	NA
39	Cyperotundone	C_15_H_22_O	1677	30.503	1.87	Sesquiterpenoid	3466-15-7
40	*epi*-α-Bisabolol	C_15_H_26_O	1685	30.808	1.06	Sesquiterpenoid	78148-59-1
41	α-Bisabolol	C_15_H_26_O	1688	30.942	0.50	Sesquiterpenoid	515-69-5
42	Selina-3,1 l-dien-9-one	C_15_H_22_O	1697	31.273	1.24	Sesquiterpenoid	NA
43	Rotundone	C_15_H_22_O	1701	31.419	0.88	Sesquiterpenoid	18374-76-0
44	Valerenol	C_15_H_24_O	1708	31.63	3.54	Sesquiterpenoid	101628-22-2
45	Selina-3,11-dien-9-ol	C_15_H_24_O	1715	31.87	1.66	Sesquiterpenoid	NA
46	Selina-4,11-dien-14-oic acid	C_15_H_22_O_2_	1721	32.082	0.51	Sesquiterpenoid	NA
47	α-Costol	C_15_H_22_O	1727	32.259	0.73	Sesquiterpenoid	65018-15-7
48	Selina-3,11-dien-9-al	C_15_H_22_O	1737	32.603	2.36	Sesquiterpenoid	NA
49	9,l l-Eremophiladien-8-one	C_15_H_22_O	1744	32.857	0.72	Sesquiterpenoid	NA
50	Aristolone	C_15_H_22_O	1749	33.023	0.70	Sesquiterpenoid	6831-17-0
51	Guaia-1 (10),11-dien-9-one	C_15_H_22_O	1752	33.113	0.21	Sesquiterpenoid	NA
52	Dehydrofukinone	C_15_H_22_O	1769	33.693	3.83	Sesquiterpenoid	19598-45-9
53	Sinenofuranol	C_14_H_24_O_2_	1783	34.196	0.86	Sesquiterpenoid	NA
54	Dihydrokaranone	C_15_H_22_O	1791	34.481	0.24	Sesquiterpenoid	NA
55	Guaia-1(10),11-dien-15-oic acid	C_15_H_22_O_2_	1815	35.278	0.46	Sesquiterpenoid	NA
56	*oxo*-Agarospirol	C_15_H_24_O_2_	1838	35.995	0.83	Sesquiterpenoid	NA
57	Methyl palmitate	C_17_H_34_O_2_	1899	38.016	0.18	Other compounds	112-39-0
58	Dihydrocolurnellarin	C_15_H_22_O_2_	1907	38.262	0.52	Sesquiterpenoid	66873-38-9
59	1,5-Diphenyl-3-pentanone	C_17_H_18_O	1964	40.03	0.16	Other compounds	5396-91-8

(NA—Not available).

The most abundant compound identified in AEO was allo-aromadendrene (13.04%), followed by dihydro-eudesmol (8.81%), α-eudesmol (8.48%), bulnesol (7.63%), τ-cadinol (4.95%) dehydrofukinone (3.83%), valerenol (3.54%), 10-*epi*-γ-eudesmol (3.07%), cis-nerolidol (2.75%), agarospirol (2.72%), dehydrojinkoh-eremol (2.53%), selina-3,11-dien-9-al (2.36%), guaiol (2.12%), caryophyllene oxide (2.00%), cyperotundone (1.87%), *nor*-Ketoagarofuran (1.75%), selina-3,11-dien-9-ol (1.66%), γ-eudesmol (1.42%), hinesol (1.41%), selina-3,1 l-dien-9-one (1.24%), jinkoh-eremol (1.21%), β-eudesmol (1.14%), kusunol (1.07%), and *epi*-α-Bisabolol (1.06%), all of which except allo-aromadendrene (sesquiterpenes) belong to sesquiterpenoids according to their structural characteristics (Table 1). Major compounds detected in this study were consistent with those found in previously published works [13,32,38,39]. Other common compounds that were detected belong to aromatic compounds. One study reported that the predominant compounds in the essential oil of *A*. *sinensis* were sesquiterpenes/sesquiterpenoids (63.4% and 58%, for natural and fungal inoculated agarwood, respectively) [39]. The differences in the chemical composition can arise due to several factors such as geographic origin, age of the tree, hydrodistillation parameters, induction methods and post-harvest handling. Most of these compounds exhibit various pharmacological activities [38]. According to literature, allo-aromadendrene contributes to the characteristic woody odor of AEO, while agarospirol gives out the smell of spicy, peppery, and woody [40–42]. While most essential oils are volatile, AEO is relatively stable at room temperature. A study done by Syameera et al. [43] showed that the AEO is thermally stable at temperature of 80°C at 5-min and 10-min time points, without causing major loss in its terpene compounds, including monoterpenes, diterpenes sesquiterpenes, and sesquiterpenoids.

Different studies have highlighted different chemical constituents as the marker compounds for classifying the AEO. Ismail et al. [44] concluded 10-epi-ϒ-eudesmol, aromadendrane, β-agarofuran, α-agarofuran and γ-eudesmol as the marker compounds in high quality agarwood oils derived from *A*. *malaccensis*. The presence of four characteristic compounds (benzylacetone, agarospirol, aristolene, and guaiol) has been used as quality indicators for AEO (*A*. *sinensis*) in a study conducted by Tian et al. [38]. The majority of these compounds, except benzylacetone and aristolene, were present in substantial quantities in the AEO. In addition, there are no contaminants, namely dibutyl phthalate (DBP) and di(ethylhexyl) phthalate (DEHP), detected in the AEO, further ensuring that the studied sample is a high-value fragrant material. Nevertheless, as of now, there is no globally recognized standard for agarwood oil (AEO). The current grading system for AEO, which considers factors such as country-of-origin, aroma, duration of fragrance, color, thickness, and density, heavily relies on consumer perception and preference [45].

In addition, 2-(2-phenylethyl)chromone is the major aromatic constituent in agarwood, which has been extensively reported [32,46]. However, no chromone compounds, as well as their derivatives, were detected in the present study. They are rarely identified by GC-MS but are more commonly detected via LC-MS/MS [32,46].

### Brine shrimp lethality test (BSLT)

**Fig 4:**
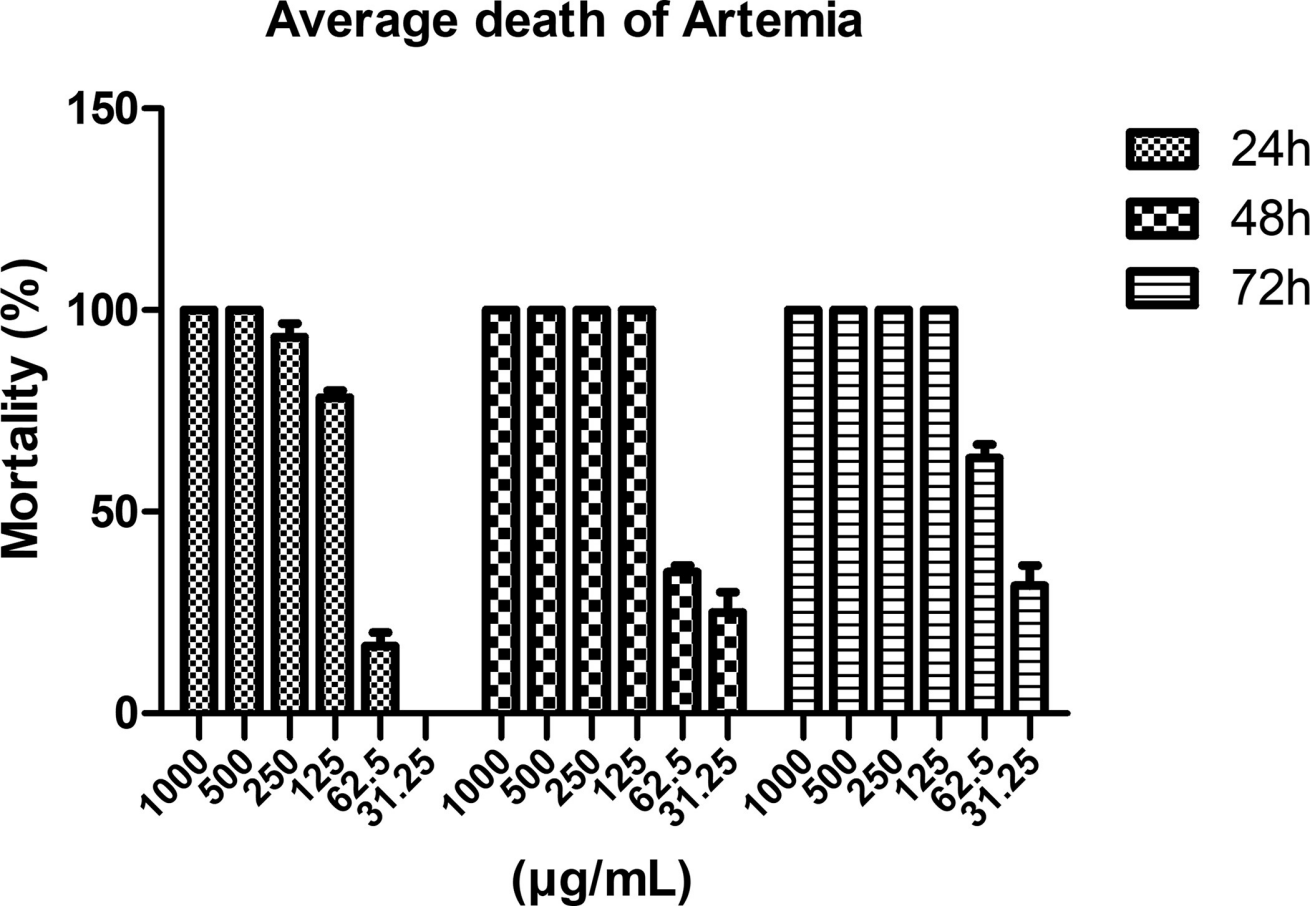
Evaluation of acute toxicity effect of AEO on brine shrimp at different treatment time-points.

The BSLT method has been widely used by researchers to assess the cytotoxic concentration range and toxicity of essential oils. The LC_50_ values obtained from brine shrimp lethality bioassay for AEO were 111.35, 66.93 and 54.05 μg/mL, for 24, 48 and 72 h, respectively (Table 2). The LC_50_ values were assessed at 95% confidence using probit analysis. The valorized essential oils should be considered toxic or medium to highly toxic by comparison to Meyer’s and Clarkson’s toxicity index as summarized in Table 2. The solvent group showed little toxicity towards brine shrimp with a survival rate of more than 90%. In contrast, AEO exhibited >90% mortality in the concentration range of 250–1000 μg/mL (Fig 4).

**Table 2:** LC_50_ value of AEO on brine shrimp lethality test (BSLT).

Time-point	LC_50_ (μg/mL)	95% confidence interval	Toxicity class (Meyer/Clarkson)
24 h	111.35	101.15–121.54	Toxic/Medium Toxic
48 h	66.93	53.77–87.42	Toxic/ Highly Toxic
72 h	54.05	45.60–66.49	Toxic/ Highly Toxic

Elumba et al. [47], Del Socorro et al. [48] suggested that some of the tested extracts with LC_50_ below 100 µg/mL which are categorized as toxic, does not always indicate its danger or out-right toxicity toward humans, but may also suggest a potential anti-tumor or anti-cancer activity [49]. To the best of our knowledge, this is the first report on the toxicity effect of AEO in brine shrimp. We hypothesized that the lethality of brine shrimp larvae may be linked to the presence of bioactive compounds in the AEO, which can interfere with biochemical and physiological processes [50]. The toxic compounds can enter the digestive tracts of brine shrimp larvae, where the substances absorbed have the ability to interact with targets, such as lipids, enzymes, cell membranes, and nucleic acids, affecting the body’s processes and ultimately resulting in death [51,52].

### Cell line viability test (MTT)

**Fig 5:**
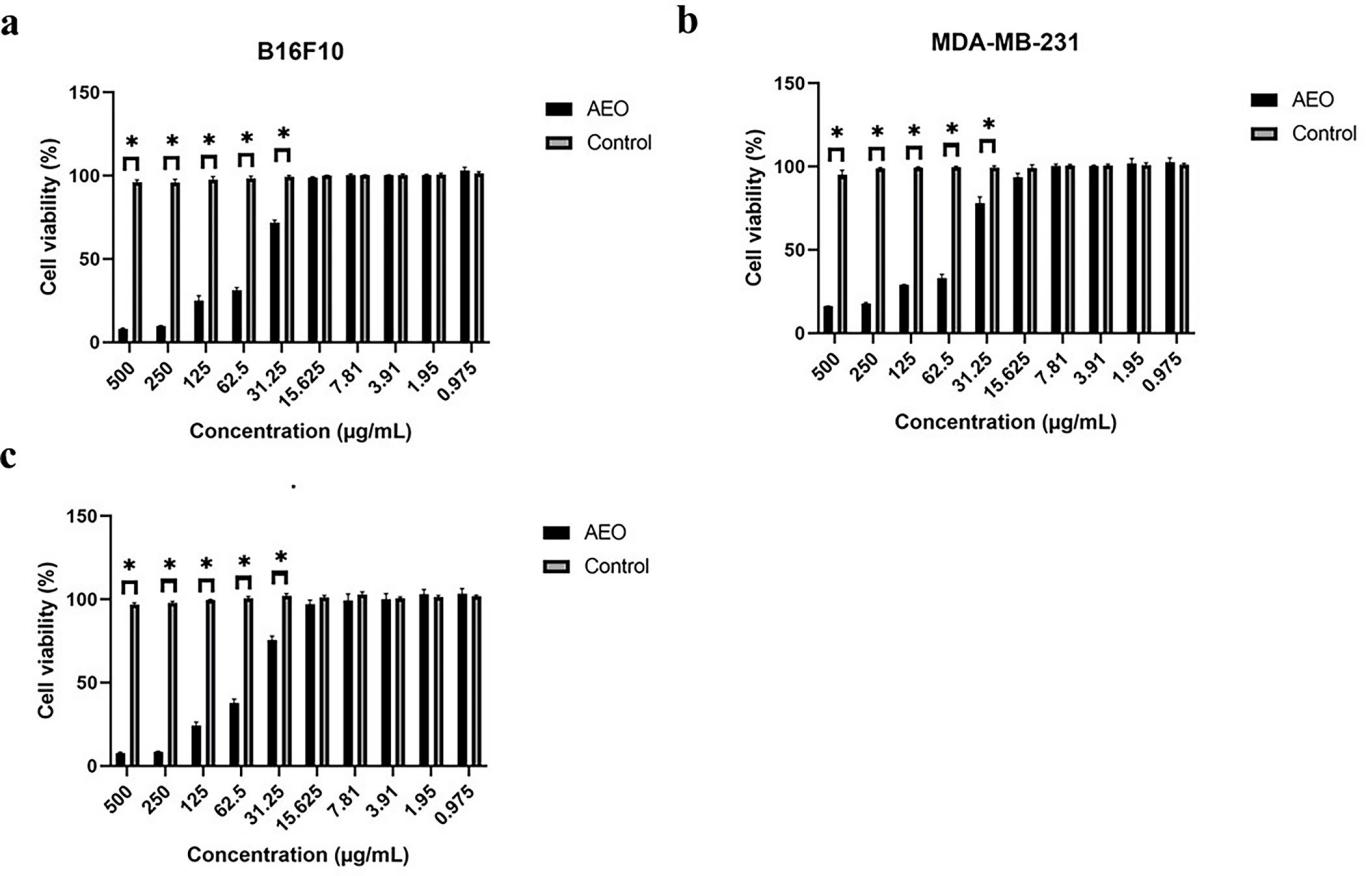
Evaluation of effect of AEO on cancer cell lines by MTT assay. Percentages of viable cells of (**A**) B16F10; (**B**) MDA-MB-231; (**C**) HepG2 at 24 h time-point after AEO treatments. Each experiment was repeated for a minimum of 3 times and plotted as bar graphs with error bars. Two-way ANOVA was conducted and the p value was calculated between control and AEO treated samples, where, *: p value < 0.05.

**Fig 6:**
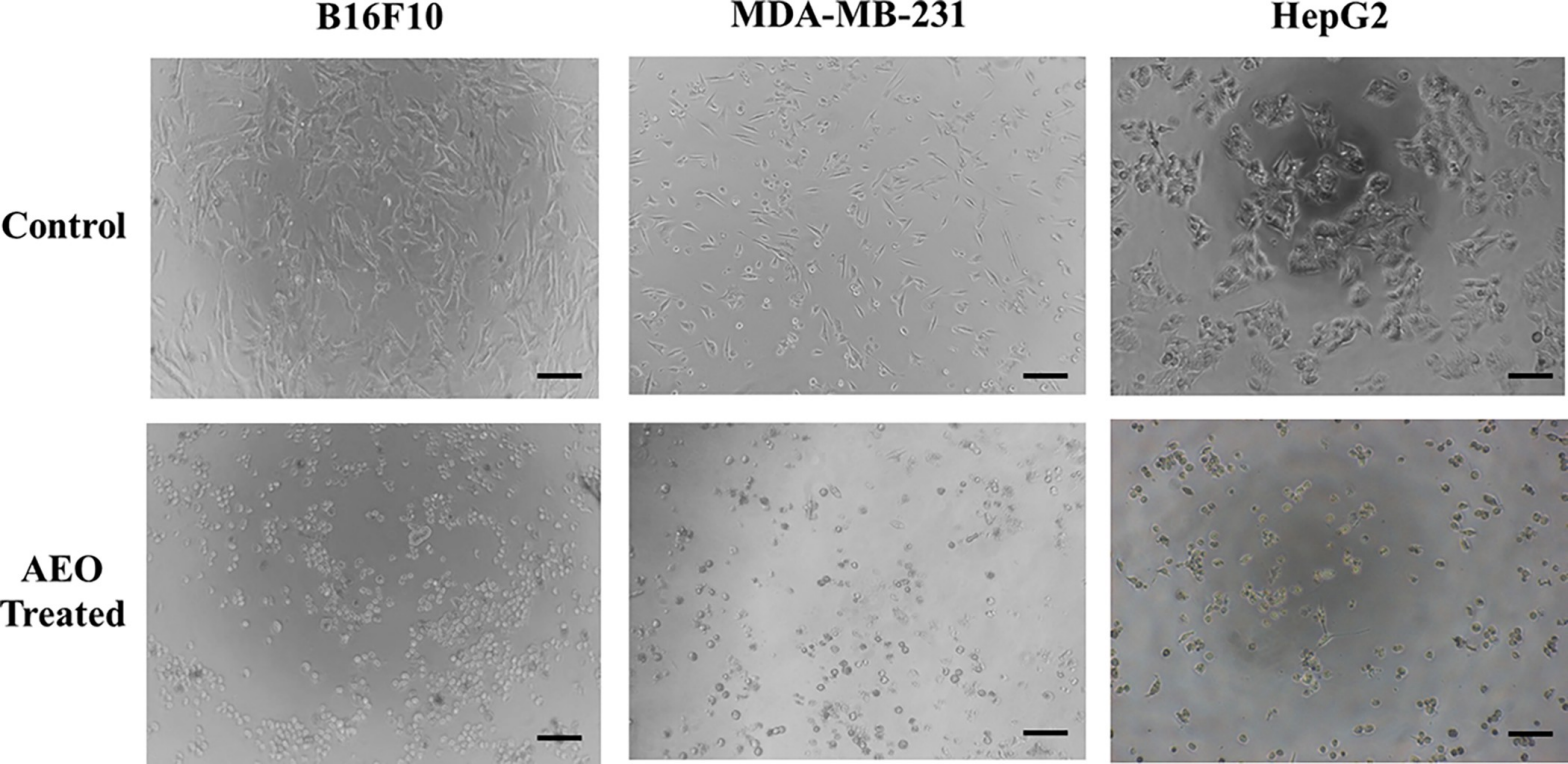
Morphology changes of B16F10, MDA-MB-231, HepG2 cells after AEO treatment (61.5 μg/mL) for 24 h. Treated cells were rounded up and cell to cell adhesion was lost. Images showing 40 x magnification. Black bar in each image represents 100 μm.

Table 3 and Fig 5A–5C show the growth inhibitory values determined from the dose-response curves of AEO and anti-cancer drugs used in cell lines MDA-MB-231, B16F10 and HepG2. The strongest anti-proliferative activity of AEO was observed against B16F10 cell line (IC_50_ 48.9 ± 3.1 μg/mL), followed by HepG2 cells (IC_50_ 56.2 ± 3.9 μg/mL) and MDA-MB-231 cell line (IC_50_ 61.3 ± 3.2 μg/mL) after 24 h treatment. As shown in Fig 6, morphological analysis revealed that AEO-induced cell rounding, loss of cell-cell contact and cell detachment, an appearance normally seen in cells undergoing apoptosis. Nevertheless, AEO treatment at 48 and 72 h also showed growth inhibitory potential on all tested cancer cell-lines. These results suggest that AEO induced a time and dose-dependent inhibition of cell proliferation, although each cell-line showed different sensitivity, in accordance with the determined IC_50_. In addition, the anti-proliferative effect of the referent chemo-drug, doxorubicin is stated in Table 3.

**Table 3:** IC_50_ values of AEO and doxorubicin (Dox) on B16F10, MDA-MB-231 and HepG2 cell lines at different treatment time-points.

Cell line	AEO IC_50_ (μg/mL)			Dox IC_50_ (μg/mL)		
	24 h	48 h	72 h	24 h	48 h	72 h
**B16F10**	48.9 ± 3.1	38.7 ± 5.0	30.5 ± 2.6	<10	<10	<5
**MDA-MB-231**	61.3 ± 3.2	54.4 ± 4.8	39.4 ± 3.7	<10	<10	<5
**HepG2**	56.2 ± 3.9	41.6 ± 2.4	36.8 ± 1.1	<10	<10	<5

At present, there is no study on AEO extracted from agarwood used against melanoma and hepatic cancer. Previous study reported that *A*. *crassna* essential oil mixture showed significant anti-proliferation activity against human cancer cell lines such as colorectal HCT 116 (IC_50_ 28 µg/mL), pancreatic PANC-1 (IC_50_ 32 µg/mL), prostate PC3 (IC_50_ 79 µg/mL) and breast MCF-7 (IC_50_ 110 µg/mL) [53]. The same group of researchers also reported that *A*. *crassna* essential oil exhibited potent cytotoxic activity against human pancreatic cancer MIA PaCa-2 cells with an IC_50_ (11 ± 2.18 μg/mL) [54]. They showed that cell migration and the colony formation ability of MIA PaCa-2 was effectively inhibited at 5 and 10 μg/mL, respectively. Furthermore, they discovered that AEO exhibited significant inhibition of HCT 116 colorectal cancer cell-induced subcutaneous tumors in nude mice [54]. It is interesting to note that these studies reported previously were using AEO derived from *A crassna*. Another scientific investigation found that AEO possesses anti-proliferative activity towards MCF-7 breast cancer cells with IC_50_ observed at 44 μg/mL [55]. However, the AEO used in this study was purchased directly from a Malaysian market, and it was unclear which type of agarwood they were using to produce AEO.

Recently, Zhang et al. [56] reported the cytotoxic activity of AEO derived from steam distillation (SD) and fungal fermentation-assisted SD (FFSD) on four different cancer-lines (human breast cancer MDA-MB-435, mouse gastric cancer MFC, human glioblastoma SNB19, mouse colon cancer MC38). They postulated that α-costal, aristolone, 10-epi-γ-eudesmol, γ-eudesmol, agarospirol, hinesol, guaiol, β-santalol, and 6-isopropenyl-4,8a-dimethyl-1,2,3,5,6,7,8,8a-octahydro- naphthalen-2-ol were antitumor ingredients of AEO. Interestingly, some of these compounds (aristolone, 10-epi-γ-eudesmol, γ-eudesmol, agarospirol, hinesol, guaiol) can be found in our AEO.

Besides sesquiterpenes, chromones, such as 2-(2-hydroxy-2-phenylethyl)chromone, demonstrated the ability to inhibit tumor development at non-cytotoxic concentrations, displaying half-inhibitory values ranging from 25 to 38 µM against different tumor cell lines [57]. Two novel chromone derivatives, namely 7-hydroxy-2-[2-(3’-methoxy-4’-hydroxyphenyl)-ethyl]chromone and 6,7-dimethoxy-2-[2-(3’-hydroxyphenyl)-ethyl]chromone, isolated from agarwood ethanol extract, exhibited weak cytotoxic activities (IC_50_: 18.82–37.95 mg/mL) against SMMC-7721, MGC-803, and OV-90 cell lines [58].

### Apoptosis assessment

**Fig 7:**
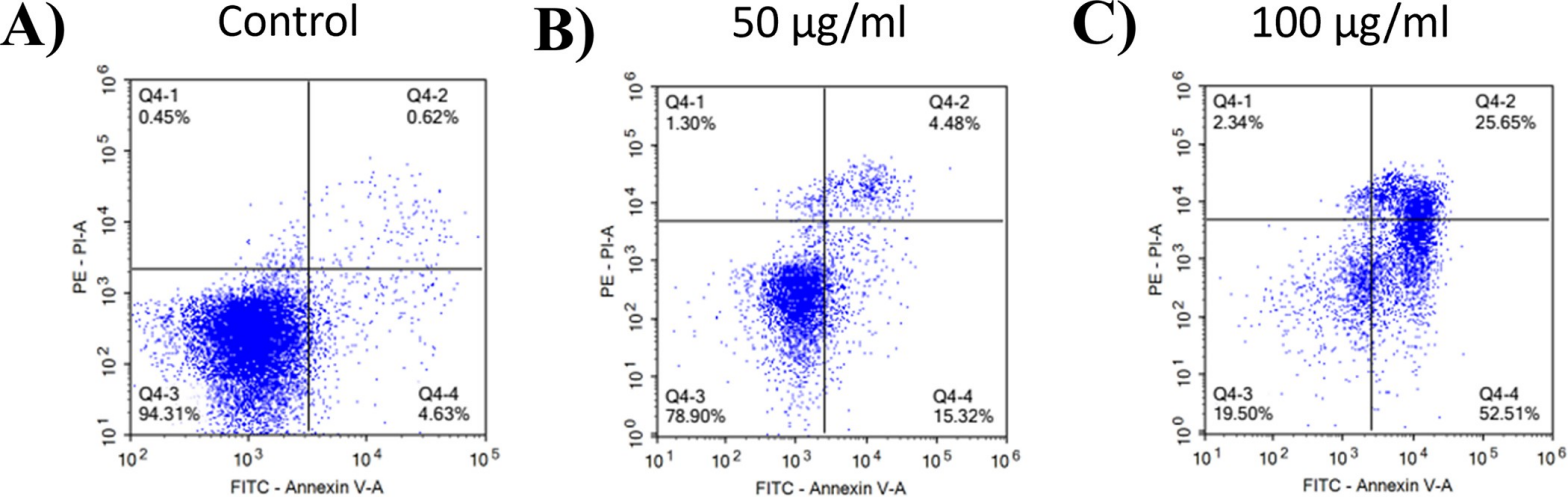
Effect of AEO on the induction of apoptosis. HepG2 cells were cultured with (A) control solvent; (B) 50 μg/mL of AEO; (C) 100 μg/mL of AEO for 16 h. Treated cells were then stained with Annexin V and PI, followed by flow cytometry analysis.

To investigate the anti-proliferative activity of AEO, treated HepG2 cells were stained with Annexin V and PI to examine the mechanism of cell death (necrosis and/or apoptosis). The populations of cells residing in the Annexin V+/PI- and the Annexin V+/PI+ quadrants were classified as early and late apoptotic cells, respectively. The Annexin V−/PI—and the Annexin V−/PI+ quadrants were designated as viable cells and necrotic cells, respectively. Control solvent treated cells showed the percentages of the apoptotic cells as < 5% after 16 h of incubation (Fig 7A). AEO-treated HepG2 samples showed varying degrees of percentages (20%-78%) of apoptotic cells (Fig 7B and 7C) (the percentages of the early apoptotic cells and the late apoptotic cells combined). Our results showed that AEO-induced apoptotic cell death in a dose-dependent manner. Recent evidence showed that activation of the apoptosis pathway is an effective strategy for cancer treatment [59]. Nevertheless, researchers have resorted to the use of essential oils as natural bioactive compounds that can elicit sensitive growth inhibition and death in cancer cells in the hope to find a more effective anticancer and chemopreventive methods [60–62].

The anticancer properties of essential oils can be attributed to their bioactive compounds and their effects on specific pathways involved in cancer cell growth and proliferation [63]. For example, bioactive compounds such as terpenes, phenolics, and other aromatic molecules may induce apoptosis, inhibit angiogenesis, or suppress the expression of genes involved in cancer progression. One study showed the growth inhibitory effect of AEO is attributed to the presence of sesquiterpene such as β-caryophyllene [53]. This compound has been shown to inhibit colorectal cancer cell proliferation, clonality, migration, invasion, and spheroid formation [53]. Further investigation revealed that the cell death mechanism induced by β-caryophyllene is related to the apoptotic properties such as DNA fragmentation and mitochondrial pathways. Moreover, studies show that volatility can be managed through appropriate formulation and application methods, allowing for controlled delivery and efficacy in anticancer treatments [64]. Research is ongoing to improve the delivery and stability of essential oils for therapeutic purposes [65]. Techniques such as encapsulation or nanoemulsion have been explored to enhance the stability and bioavailability of essential oil components [66]. Nevertheless, essential oils are often used in combination with other therapies (complementary medicine) rather than as standalone treatments [65]. These approaches can enhance their potential benefits while minimizing concerns about volatility.

## Conclusion

The AEO was successfully extracted from agarwood (*A*. *sinensis*) powder using HD. The presence of key quality marker compounds and absence of contaminants suggest that the extracted AEO is of high purity. This study demonstrated for the first time the cytotoxic effects of AEO on brine shrimps as well as melanoma (B16F10), hepatocarcinoma (HepG2) and breast adenocarcinoma (MDA-MB-231) cancer cell lines. We showed that the anti-proliferative activity of the AEO is time and dose-dependent. Furthermore, our results indicated that the cytotoxic or growth inhibitory effect may be attributable to the presence of sesquiterpene and sesquiterpenoid, which is capable of inducing apoptotic cell death. Nevertheless, we also observed a positive correlation between BSLT and *in vitro* testing of cancer cell lines. Future studies should be directed to elucidate the cytotoxic agent(s) as well as the anti-cancer mechanism of AEO in order to enhance understanding and position the essential oil as a potential cancer remedy.

## Supporting information

S1 File Chemicals and reagents used in this study. Dulbecco’s Modified Eagle’s medium (DMEM), trypsin, phosphate buffer saline (PBS) and fetal bovine serum (FBS) were purchased from Gibco, UK. Propylene glycol (PEG), standard drug doxorubicin (Dox) and 3-(4,5-dimethylthiazol-2-yl)-2-5-diphenyltetrazolium bromide (MTT) reagent were procured from Sigma-Aldrich, USA. Tween 80 was obtained from Acros Organics, USA.
(DOCX)

S2 File Link to dataset. Toxicity data of AEO on brine shrimp at different treatment time-points. https://osf.io/pfwba/?view_only=f5b419159a024c97b463ef6d4c4b9626.
(DOCX)

S3 File Link to dataset. Cell viability of B16F10 melanoma, MDA-MB-231 breast, HepG2 hepatocarcinoma cell-lines after 24h treatment and the IC50 data for 24h, 48h, 72h treatment. https://osf.io/tcm86/?view_only=77247d8859594c8cb9d53787db03661a.
(DOCX)

S4 File Link to data analysis. Statistical analysis of MTT data. https://osf.io/hw59q/?view_only=dde0aa11171847e58fb183460c93dd88.
(DOCX)

S5 File Link to dataset. Flow cytometry results of AEO treated HepG2 cells. https://osf.io/pywq7/?view_only=0d6a63a87f784b37b4e7aedfa6faf263.
(DOCX)

S6 File (DOCX)

S7 File (PDF)
