# Supplementary material for: Chemical profiling and cytotoxicity screening of agarwood essential oil (Aquilaria sinensis) in brine shrimp nauplii and cancer cell lines
Source: PLoS One. 2024 Nov 7;19(11):e0310770. doi: 10.1371/journal.pone.0310770 (PMC11542896; doi:10.1371/journal.pone.0310770)
Supplement: S1 File — Dulbecco’s Modified Eagle’s medium (DMEM), trypsin, phosphate buffer saline (PBS) and fetal bovine serum (FBS) were purchased from Gibco, UK. Propylene glycol (PEG), standard drug doxorubicin (Dox) and 3-(4,5-dimethylthiazol-2-yl)-2-5-diphenyltetrazolium bromide (MTT) reagent were procured from Sigma-Aldrich, USA. Tween 80 was obtained from Acros Organics, USA. (DOCX) [file pone.0310770.s001.docx]

**Supporting Information**

**S1 Chemicals and reagents used in this study.** Dulbecco’s Modified Eagle’s medium (DMEM), trypsin, phosphate buffer saline (PBS) and fetal bovine serum (FBS) were purchased from Gibco, UK. Propylene glycol (PEG), standard drug doxorubicin (Dox) and 3-(4,5-dimethylthiazol-2-yl)-2-5-diphenyltetrazolium bromide (MTT) reagent were procured from Sigma-Aldrich, USA. Tween 80 was obtained from Acros Organics, USA.
