# Supplementary material for: Chemical profiling and cytotoxicity screening of agarwood essential oil (Aquilaria sinensis) in brine shrimp nauplii and cancer cell lines
Source: PLoS One. 2024 Nov 7;19(11):e0310770. doi: 10.1371/journal.pone.0310770 (PMC11542896; doi:10.1371/journal.pone.0310770)
Supplement: S2 File — Toxicity data of AEO on brine shrimp at different treatment time-points. https://osf.io/pfwba/?view_only=f5b419159a024c97b463ef6d4c4b9626. (DOCX) [file pone.0310770.s002.docx]

**Supporting Information**

**S2 File 1** Toxicity data of AEO on brine shrimp at different treatment time-points.

<https://osf.io/pfwba/?view_only=f5b419159a024c97b463ef6d4c4b9626>
