# Supplementary material for: Chemical profiling and cytotoxicity screening of agarwood essential oil (Aquilaria sinensis) in brine shrimp nauplii and cancer cell lines
Source: PLoS One. 2024 Nov 7;19(11):e0310770. doi: 10.1371/journal.pone.0310770 (PMC11542896; doi:10.1371/journal.pone.0310770)
Supplement: S3 File — Cell viability of B16F10 melanoma, MDA-MB-231 breast, HepG2 hepatocarcinoma cell-lines after 24h treatment and the IC50 data for 24h, 48h, 72h treatment. https://osf.io/tcm86/?view_only=77247d8859594c8cb9d53787db03661a. (DOCX) [file pone.0310770.s003.docx]

**Supporting Information**

**S3 File 2** Cell viability of B16F10 melanoma, MDA-MB-231 breast, HepG2 hepatocarcinoma cell-lines after 24h treatment and the IC50 data for 24h, 48h, 72h treatment.

<https://osf.io/tcm86/?view_only=77247d8859594c8cb9d53787db03661a>
