# Supplementary material for: Chemical profiling and cytotoxicity screening of agarwood essential oil (Aquilaria sinensis) in brine shrimp nauplii and cancer cell lines
Source: PLoS One. 2024 Nov 7;19(11):e0310770. doi: 10.1371/journal.pone.0310770 (PMC11542896; doi:10.1371/journal.pone.0310770)
Supplement: S4 File — Statistical analysis of MTT data. https://osf.io/hw59q/?view_only=dde0aa11171847e58fb183460c93dd88. (DOCX) [file pone.0310770.s004.docx]

**Supporting Information**

**S4 File 3** Statistical analysis of MTT data.

<https://osf.io/hw59q/?view_only=dde0aa11171847e58fb183460c93dd88>
