# Supplementary material for: Chemical profiling and cytotoxicity screening of agarwood essential oil (Aquilaria sinensis) in brine shrimp nauplii and cancer cell lines
Source: PLoS One. 2024 Nov 7;19(11):e0310770. doi: 10.1371/journal.pone.0310770 (PMC11542896; doi:10.1371/journal.pone.0310770)
Supplement: S5 File — Flow cytometry results of AEO treated HepG2 cells. https://osf.io/pywq7/?view_only=0d6a63a87f784b37b4e7aedfa6faf263. (DOCX) [file pone.0310770.s005.docx]

**Supporting Information**

**S5 File 4** Flow cytometry results of AEO treated HepG2 cells.

<https://osf.io/pywq7/?view_only=0d6a63a87f784b37b4e7aedfa6faf263>
