# Supplementary material for: Chemical profiling and cytotoxicity screening of agarwood essential oil (Aquilaria sinensis) in brine shrimp nauplii and cancer cell lines
Source: PLoS One. 2024 Nov 7;19(11):e0310770. doi: 10.1371/journal.pone.0310770 (PMC11542896; doi:10.1371/journal.pone.0310770)
Supplement: S6 File — (DOCX) [file pone.0310770.s006.docx]

**Supporting Information**

**S1 Chemicals and reagents used in this study.** Dulbecco’s Modified Eagle’s medium (DMEM), trypsin, phosphate buffer saline (PBS) and fetal bovine serum (FBS) were purchased from Gibco, UK. Propylene glycol (PEG), standard drug doxorubicin (Dox) and 3-(4,5-dimethylthiazol-2-yl)-2-5-diphenyltetrazolium bromide (MTT) reagent were procured from Sigma-Aldrich, USA. Tween 80 was obtained from Acros Organics, USA.

**S2 File 1** Toxicity data of AEO on brine shrimp at different treatment time-points.

<https://osf.io/pfwba/?view_only=f5b419159a024c97b463ef6d4c4b9626>

**S3 File 2** Cell viability of B16F10 melanoma, MDA-MB-231 breast, HepG2 hepatocarcinoma cell-lines after 24h treatment and the IC50 data for 24h, 48h, 72h treatment.

<https://osf.io/tcm86/?view_only=77247d8859594c8cb9d53787db03661a>

**S4 File 3** Statistical analysis of MTT data.

<https://osf.io/hw59q/?view_only=dde0aa11171847e58fb183460c93dd88>

**S5 File 4** Flow cytometry results of AEO treated HepG2 cells.

<https://osf.io/pywq7/?view_only=0d6a63a87f784b37b4e7aedfa6faf263>
