# Supplementary material for: Chemical profiling and cytotoxicity screening of agarwood essential oil (Aquilaria sinensis) in brine shrimp nauplii and cancer cell lines
Source: PLoS One. 2024 Nov 7;19(11):e0310770. doi: 10.1371/journal.pone.0310770 (PMC11542896; doi:10.1371/journal.pone.0310770)
Supplement: S7 File — (PDF) [file pone.0310770.s007.pdf]

Supporting Information

Chemicals and reagents

Dulbecco's Modified Eagle's medium (DMEM), trypsin, phosphate buffer saline (PBS) and fetal bovine serum (FBS) were purchased from Gibco, UK. Propylene glycol (PEG), standard drug doxorubicin (Dox) and 3-(4,5-dimethylthiazol-2-yl)-2-5-diphenyltetrazolium bromide (MTT) reagent were procured from Sigma-Aldrich, USA. Tween 80 was obtained from Acros Organics, USA.

S1 Toxicity data of AEO on brine shrimp at different treatment time-points.

https://osf.io/pfwba/?view_only=f5b419159a024c97b463ef6d4c4b9626

S2 Cell viability of B16F10 melanoma, MDA-MB-231 breast, HepG2 hepatocarcinoma cell-lines after 24h treatment and the IC50 data for 24h, 48h, 72h treatment.

https://osf.io/tcm86/?view_only=77247d8859594c8cb9d53787db03661a

S3 Statistical analysis of MTT data.

https://osf.io/hw59q/?view_only=dde0aa11171847e58fb183460c93dd88

S4 Flow cytometry results of AEO treated HepG2 cells.

https://osf.io/pywq7/?view_only=0d6a63a87f784b37b4e7aedfa6faf263
